# Effects of Community-Based Natural Resource Management on Household Welfare in Namibia

**DOI:** 10.1371/journal.pone.0125531

**Published:** 2015-05-12

**Authors:** Brianne Riehl, Hisham Zerriffi, Robin Naidoo

**Affiliations:** 1 Department of Earth, Ocean and Atmospheric Sciences, University of British Columbia, Vancouver, B.C., Canada; 2 Institute for Resources, Environment, and Sustainability, University of British Columbia, Vancouver, B.C., Canada; 3 WWF-US, 1250 24th Street NW, Washington, D.C., United States of America; University of Waterloo, CANADA

## Abstract

Biodiversity conservation, as an environmental goal, is increasingly recognized to be connected to the socioeconomic well-being of local communities. The development of a widespread community-based natural resource management (CBNRM) program in Namibia makes it an ideal location to analyze the connection between conservation and socioeconomic well-being of local communities. Namibia’s CBNRM program involves the formation of communal conservancies within rural communities and previous studies have found it to be successful on both ecological and economic fronts. In order to broaden the understanding of the program’s impact to include social factors, we have conducted a comparative analysis to determine the effects of this program on household welfare outcomes. Data from two rounds of the Namibia Demographic and Health Surveys (2000 and 2006/07) and quasi-experimental statistical methods were used to evaluate changes in various health, education and wealth outcomes of those living in conservancies, relative to non-conservancy comparison groups. Regression results indicate mixed effects of the conservancy program at the household level. The program had positive effects on some health outcome variables, including bednet ownership, which was twice as likely to increase over time in conservancy compared to non-conservancy households. Program impacts were negative for education outcomes, with the proportion of school attendance of conservancy children being 45% less likely to increase over time than non-conservancy children. Wealth outcome results were inconclusive. Our findings highlight the importance of analyzing community conservation programs at a variety of scales when evaluating overall impact, as community-level benefits may not necessarily extend down to the household level (and vice versa).

## Introduction

Biodiversity conservation is an important area of environmental policy, and increasingly recognized to be connected to the socioeconomic well-being of local communities [1–3]. The social impact of conservation programs on communities is an intensely debated topic, fostering a wide spectrum of views regarding whether or not human development and conservation can be achieved simultaneously [1–3]. Those most vulnerable to the deterioration of natural systems are typically rural populations of the developing world, who commonly lack a minimum standard of living [4]. The strong dependence of these populations on natural resources for their livelihoods leads to a complex relationship between conservation and human development [3]. Four common perspectives on this relationship include [1]:

1. Socioeconomic development and conservation are separate policy realms
2. Socioeconomic development is a critical constraint on conservation
3. Conservation efforts should not compromise socioeconomic development
4. Socioeconomic development depends upon conservation

Despite sound reasoning and evidence to support each of these perspectives, research is increasingly citing evidence to support the final statement that the future of biodiversity conservation and the socioeconomic needs of rural communities are intricately connected [2–4]. The Millennium Ecosystem Assessment (2005) provides a compelling argument to explain the dependence of human well-being on the services provided by nature, suggesting that threats to natural assets must be addressed as part of an effective strategy for human development. Explanations for this linkage often refer to the fundamental dependence of humans on services derived from natural ecosystems, expressing an increasing concern for the potential health and welfare impacts of continued ecosystem degradation [5,6]. Ecosystems provide provisioning, regulating, cultural and supportive services, which are particularly important for rural communities who often rely directly on these services for their livelihood [4,5,7]. Links between biodiversity conservation and human well-being include food security, health improvements, income generation, reduced vulnerability to climate and resource changes, ecosystem services, and cultural value [8]. Emphasis in recent literature on these linkages suggests a momentum towards approaching environmental conservation and human development in an integrated way [2,6–8].

Previous analyses of community-based resource management initiatives have determined that, because of their knowledge and direct dependence on the land being protected, local communities can often undertake conservation more effectively and cost efficiently than a centralized government agency [2,4]. A key principle underlying these community-based initiatives is to align long-term conservation with the short-term needs of local people, ensuring that community members gain some benefit for their participation in conservation efforts [1,2]. It is important to analyze these initiatives in order to further understand the relationship between conservation and human well-being [1–3]. Examples from southern Africa suggest strong linkages between the quality of natural resources and their management, and a variety of socioeconomic and livelihoods indicators [5,9–11]. Nevertheless, rigorous empirical evaluations, even in this part of the world, remain rare [12,13].

Regardless of the academic debate, many international conservation organizations and development agencies strongly emphasize the links between rural poverty and environmental degradation in their programmatic activities [1–3,8,9]. However, the evidence base for the effectiveness of these programs in achieving their objectives is relatively sparse, particularly in the use of rigorous program evaluation techniques [14]. Here, we investigate one such existing conservation and local development program to determine whether these goals can be jointly achieved.

The specific aim is to evaluate whether an existing community-based conservation effort in Namibia can successfully contribute to improved socioeconomic well-being in local communities. Previous analyses and evaluations of Namibia’s Community-Based Natural Resource Management (CBNRM) program have focused largely on community-level benefits, often using cross-sectional and highly aggregated data with limited geographic resolution [10,15–17]. These studies have generally demonstrated positive impacts on community-level benefits and/or welfare as a result of the program. Fewer studies have examined whether these benefits are also present at the household level, and of those that have, results have been a mix of positive [12,13], negative [18] or contrasting [17] impacts. Fewer still have used quasi-experimental methods [12,13]. Our analysis here builds on work from these latter two references but assesses a broader range of welfare outcomes. We focus on households and individuals within communities to determine whether community-level benefits extend down to the household level in a variety of livelihood-related dimensions. We use matching methods from the program evaluation literature [19] to construct appropriate comparison groups between conservancy and non-conservancy households. We then use regression models that statistically compare temporal trends between conservancy and non-conservancy units on a variety of socioeconomic outcomes.

## Methods

### Study area

Namibia is a country located in southern Africa, with a population of 2.259 million people [20]. The majority of this population (62%) lives in rural areas and depends on natural resources for their livelihood [21,22]. Although ranked as a middle-income country, the distribution of income in Namibia is highly skewed with a 51% unemployment rate and a 38.2% incidence of poverty in rural areas [20,21]. Most rural Namibians generate income through farming (livestock and crop production in north-central and eastern areas, mainly livestock production in the arid north-western and southern areas) [21].

**Fig 1:**
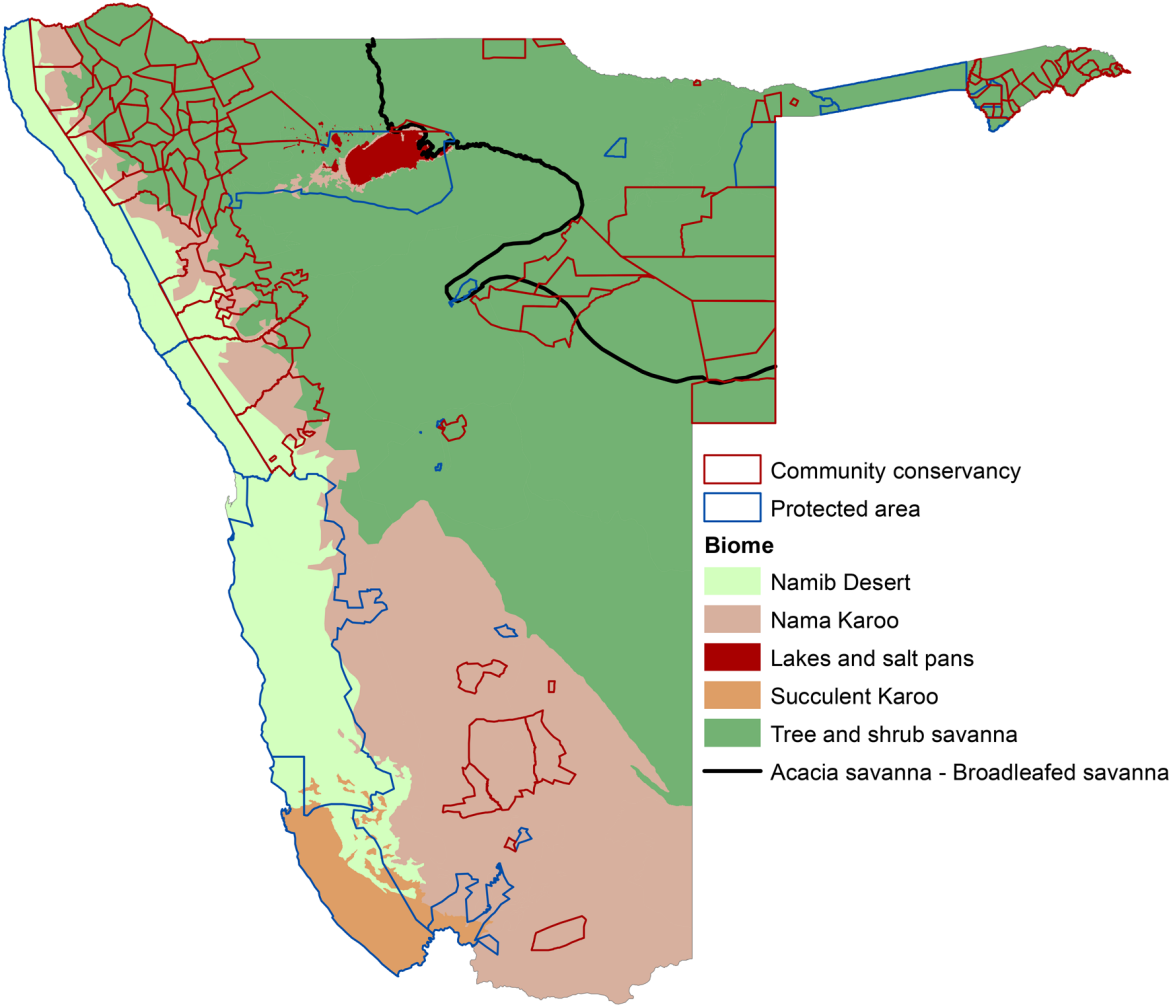
Communal conservancies and protected areas in relation to Namibia’s six major biomes [21].

The Namibian climate ranges from arid and semiarid in the west, including temperate coastal desert, to more subtropical in the northeast [23,24]. This broad range of ecosystem types can be summarized into six major biomes (Fig 1) based on similar plant life and climatic characteristics [21]. These ecosystems are home to remarkable biodiversity, including more than 4,500 plant taxa, almost 700 of which are endemic to the country, as well as 217 species of mammals, 26 of which are endemic [24]. This incredible species richness and endemism makes Namibia a critical location for conservation programs that can offer protection of this biodiversity while promoting the social and economic well-being of local communities.

### Namibia’s Community Based Natural Resource Management Program

Namibia experienced high hunting and poaching pressures which, in combination with disenfranchisement, civil war, and drought, led to a decrease in populations of many large wildlife species and large-scale animal migrations in the 1970s and 1980s [21]. Partially in response to these threats to biodiversity, the Nature Conservation Act was passed in 1996, allowing for the formation of communal conservancies, i.e., areas of customary land tenure in which rights to benefits derived from natural resources are devolved to local communities. Various community-based conservation activities had begun in 1991, but the passing of this legislation was the official beginning of Namibia’s CBNRM program [7,21,25].

By the end of 2013, the CBNRM program included 79 registered conservancies covering 19.4% of Namibia’s land surface and bringing the total land surface under conservation management to 43% [21]. These conservancies vary greatly in size, environment, human population density, wildlife resources and tourism potential, providing significant variation in income gains and challenges for conservancy management [15–18,25]. A main focus of conservancies is wildlife management, as healthy populations of indigenous wildlife are central to unlocking the value of natural resources in the region. The sustainable use of wildlife through tourism, trophy hunting and own-use consumption is particularly valuable in communal areas where agricultural land uses are limited by low, erratic rainfall and infertile soils [21]. It is recognized that these wildlife-based benefits are heavily reliant on foreign visitors to the country, and ecosystem services provided by conservancies are increasingly diversifying through the sustainable harvest of indigenous plant products, fishing, and craft sales [21,25].

**Fig 2:**
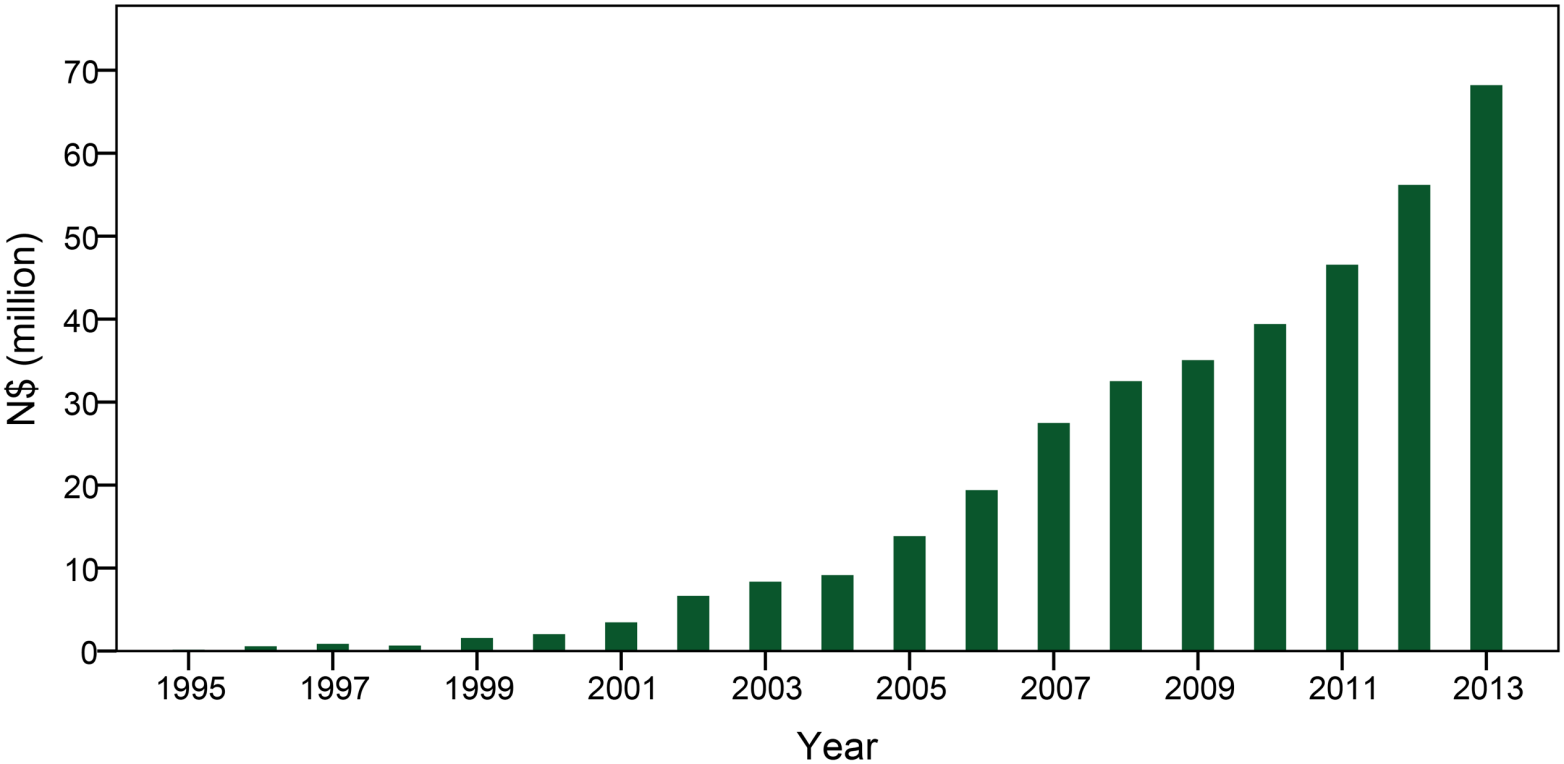
Total cash income and in-kind benefits of Namibia’s Community-Based Natural Resource Management program. Cash income includes fees paid to conservancies by tourism and hunting operators, as well as resident wages from these operations. In-kind benefits include game meat and fringe benefits provided to employees by the private sector. Note that as of 2013, 10 $N equals approximately 1 USD [21].

The CBNRM program operates with three main goals: natural resource management and conservation, rural development, and empowerment and capacity building [7,21]. The program is generally considered a success, gaining national and international recognition for making an important contribution to both environmental and socioeconomic development goals [7,21]. From 1991–2013 the program contributed N$ 3.92 billion (approximately US$ 392 million at 2013 exchange rates) in total economic value to Namibia’s net national income, with CBNRM activities generating more than N$ 68 million (US$ 6.8 million) in 2013 (Fig 2). The program has generated 6472 jobs between 1991 and 2013, as well as contributed to dramatic increases in wildlife numbers and range expansion, one example being lions (*Panthera leo*) in the northwest. Another noteworthy impact of this program has been a major attitudinal shift towards natural resources, as wildlife previously perceived as a detriment to livelihoods are increasingly seen as an asset and regarded with great pride by conservancy members [21].

Despite these examples of community-level socioeconomic benefits, there remains a level of discontent with CBNRM as a development strategy. One of the main sources of discontent is the issue of equity in the distribution of conservancy benefits, as well as arguments that the indirect benefits (such as improved infrastructure, communal soup kitchens, waterpoints, schools and clinics) expected to promote development for all conservancy residents have not yet materialized [13,18].

In this paper, we analyze a different aspect of the potential benefits of the conservancies, namely whether the direct and indirect community level benefits may also result in measurable household level benefits. Households could benefit from membership in the community conservancy in one of two ways. First, there could be benefits that end up flowing directly to the households. This could take the form of jobs that improve household wealth as well as other forms of possible benefit-sharing. Second, households could benefit from the investments made at the community level (e.g. improved schools should lead to improved educational opportunities and improved health infrastructure should result in improved health treatment). Prior assessments of the CBNRM program have tended to focus on the community investments that are made while this analysis focuses on detecting household level changes.

### Demographic and Health Surveys Data

**Fig 3:**
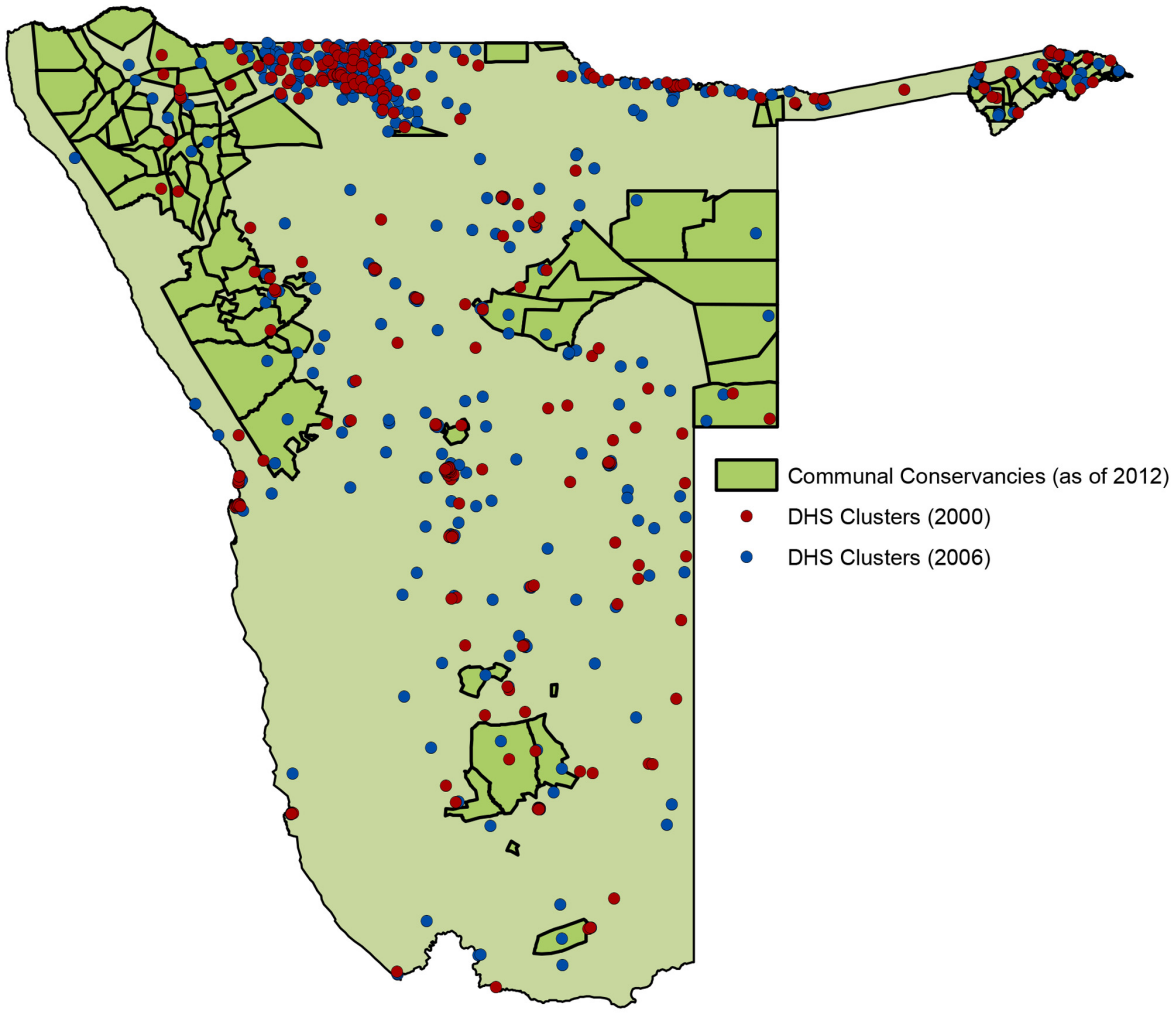
Map of DHS cluster locations for the 2000 and 2006/07 surveys.

We used Demographic and Health Surveys (DHS) data from 2000 and 2006/07 to evaluate the effect of conservancies on various health, education and wealth outcome variables. The DHS are nationally and sub-nationally representative surveys, implemented using a stratified 2-stage cluster sampling design. They contain detailed demographic and socioeconomic data at both the individual and household level, obtained by interviewing women and men aged 15–49 on a variety of issues related to household assets, reproductive health, family planning and child health. In Namibia, 6392 households participated in the 2000 survey (household response rate: 97%; individual response rate: 92%) and 9200 households participated in the 2006/07 survey (household response rate: 98%; individual response rate: 95%) [26,27]. Households were classified as in-conservancy if they were located in a conservancy that was registered prior to the 2006/07 DHS. This included 429 households in 13 conservancies in 2000 and 581 households in 22 conservancies in 2006/07 (Fig 3). DHS surveys do not provide panel data, meaning that households interviewed in 2006/07 are different than those sampled in 2000.

The DHS are globally recognized as a key source of comparative quantitative data across developing countries [28]. DHS surveys in Namibia were conducted by the Ministry of Health and Social Services (MoHSS) in collaboration with the Central Bureau of Statistics and with technical assistance provided by ICF Macro through the MEASURE DHS project [26,27]. Survey design and implementation passed a national ethics review panel and participation was voluntary with informed consent obtained from all survey respondents. Additional ethics approval by our Institutional Review Board was not necessary as it is not required for secondary data without identifying information on individuals or households.

The geographic matching variables (distance to main roads, geographical region, precipitation, altitude and biome) were determined using GIS data provided by the Namibian Association of CBNRM Support Organizations (NACSO) (www.nacso.org.na) and the Environmental Information Service (EIS)-Namibia (http://www.nnf.org.na/eis/30.html). The distance to main roads variable was calculated in meters from the geographical center of each conservancy. Households were classified as either inside or outside of conservancies based on a shapefile of communal conservancy boundaries obtained from EIS-Namibia.

### Outcome Variables of Interest

The outcome variables we chose to analyze cover four main categories related to socioeconomic well-being: (1) disease prevention, (2) disease prevalence and treatment, (3) education and (4) wealth. When choosing outcome variables, we were limited to those asked in both the 2000 and 2006/07 DHS surveys with a good response rate. The dependent variables chosen to best represent the four socioeconomic categories of interest are: bednet ownership and usage (1), diarrhea prevalence and treatment (2), school attendance (3) and wealth index (4) (S1 Table).

#### Bednet ownership and usage

These variables were chosen to represent disease prevention as an indicator of socioeconomic well-being. Malaria is the 10^th^ most common cause of death in Namibia and bednets treated with insecticide are an effective way to prevent the disease [29]. Survey respondents were asked whether the household owned a bednet, and whether she slept under a bednet the previous night. We coded households with a bednet as “1” and households without a bednet as “0” for the ownership outcome variable. For the bednet usage variable, we coded respondents who slept under the bednet as “1” and those who didn’t as “0”. It is important to note that our analysis of bednet usage was limited by the 2000 DHS dataset to female respondents only. This means that if the respondent reported no to this question, there is still the possibility that a child or other female household member used the bednet during the previous night.

#### Diarrhea prevalence and treatment

These variables represent disease prevalence and treatment as an indicator of socioeconomic well-being. Diarrheal diseases are the 5^th^ most common cause of death in Namibia and were therefore important to capture in our outcome variables [29]. In the DHS surveys, mothers of children under age 5 were asked whether their child had experienced diarrhea in the past 2 weeks. We coded children whose mothers answered yes with a “1” and the remaining children with “0”. The surveys also asked mothers who answered yes whether the child had received any treatment for this bout of diarrhea. We coded children that had received medical treatment with a “1” and those that hadn’t with a “0”.

#### School attendance

This variable represents education as an indicator of socioeconomic well-being. DHS data contains information on the school attendance of each household member. We chose school-aged children (ages 6 to 16) as our unit of analysis and coded children who were currently attending school with a “1” and all others with a “0”.

#### Wealth Index

This variable represents wealth as an indicator of socioeconomic well-being. Relative household wealth is included in DHS data as an asset-based wealth index, reported as both a standardized factor score and as a quintile. Data on household asset ownership collected during the survey are dichotomized and entered into a principal component analysis (PCA), which assigns weights to each asset. The asset values are then weighted accordingly and summed for each household, yielding the household factor score [30]. We used this standardized factor score as a continuous variable in our analysis of household wealth.

### Statistical matching methods

**Fig 4:**
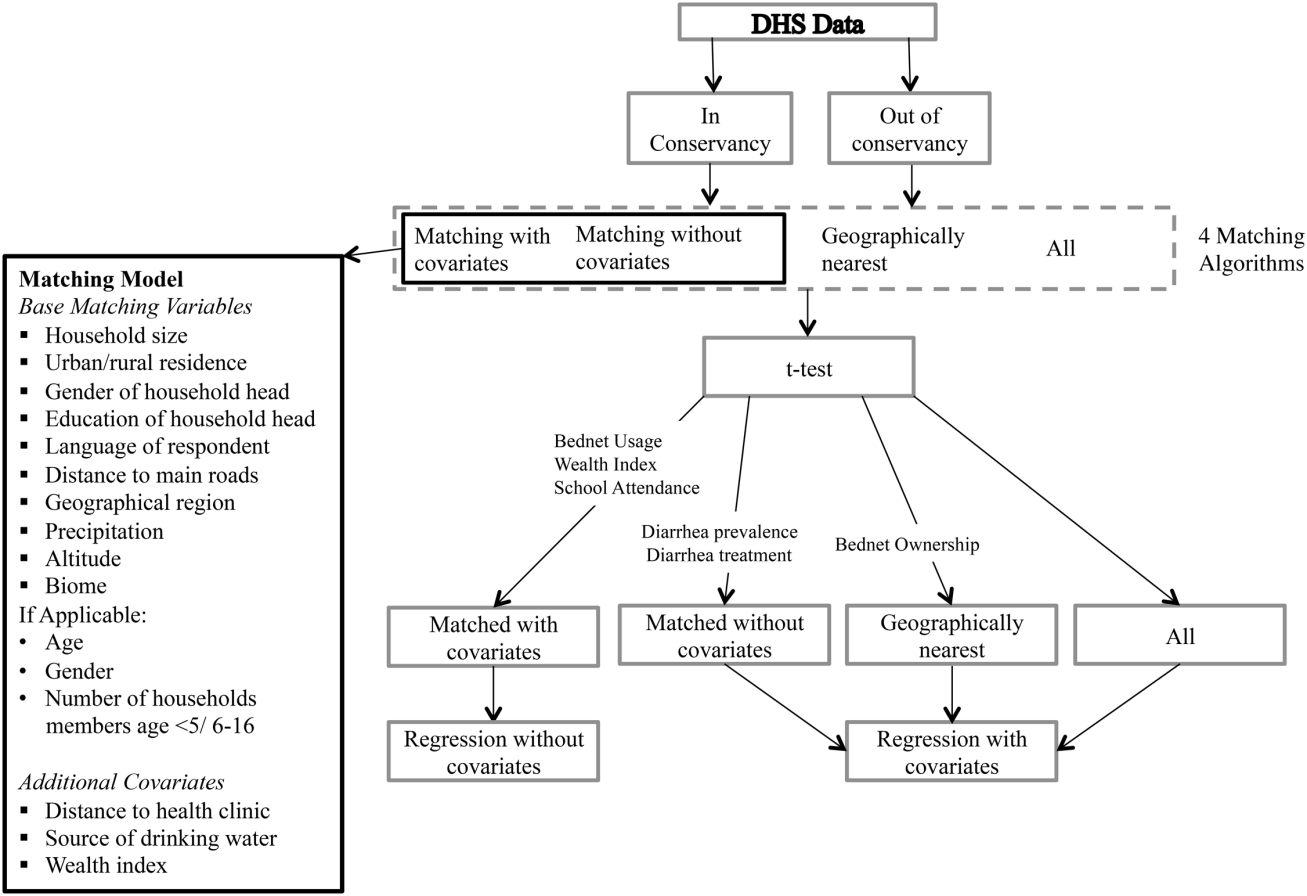
Flowchart summary of methods.

In order to compare conservancy (treatment) and non-conservancy (comparison) households/individuals, comparison groups were created with the goal of having non-statistically different treatment and comparison groups for each outcome variable in 2000. This ensures that any difference in temporal trends between the two groups can be attributed to the presence or absence of a conservancy program. Three comparison groups were created for each treatment group and were evaluated based on mean differences from the treatment group for each outcome variable. The best comparison group was chosen for each outcome variable, applied to the 2006/07 data, and then used in a regression analysis. The purpose of the regression was to determine the effect of conservancy residence over time on each socioeconomic outcome (Fig 4).

We compared trends in conservancy households with three non-conservancy comparison groups: (1) all surveyed households outside of conservancies; (2) all surveyed households in the nearest DHS sampling cluster outside of each surveyed conservancy; and (3) a matched comparison group determined using Mahalanobis distance matching [19]. A statistically matched comparison group (3) was used, since quasi-experimental matching models are regarded as one of the best alternatives when random experiment design is not possible [31]. This comparison group (3) was matched to be similar to in-conservancy households in terms of characteristics that may confound the conservancy impact on socioeconomic indicators of interest. The variables of socioeconomic well-being we were interested in analyzing included both household level variables and individual level variables. For this reason, the three comparison groups were created for four different units of analysis: (a) households; (b) children under 5; (c) school-aged children; and (d) female respondents.

For the matched comparison group (3), conservancy households/individuals were matched with households/individuals outside of conservancies using the following variables for all four units of analysis:

1. Number of household members
2. Urban/rural residence
3. Gender of household head
4. Education of household head
5. Language of household respondent
6. Distance to main roads
7. Geographical region
8. Precipitation
9. Altitude
10. Biome

Variables used only for the individual units of analysis are as follows:

1. Age (b, c, d)
2. Gender (b, c)
3. Number of household members aged 5 and under (b)
4. Number of household members between 6 and 16 years of age (c)

Some variables were used in the matching model for only the relevant outcome variables. These include:

1. Distance to nearest health clinic
2. Source of drinking water
3. Wealth Index

Distance to a health clinic was used only for outcome variables related to health and disease, source of drinking water only for diarrhea-related outcome variables, and household wealth index was included only for health and education outcome variables. Matching variables chosen are those that may determine both economic and social well-being as they can affect access to economic opportunities, health care and education.

The matching model used in the creation of the matched comparison groups (3) was 1-to-1 nearest neighbour matching with replacement, using a Mahalanobis distance metric. This model was found to be the best at producing comparison groups with matching variable distributions that were similar to those of the treatment (conservancy) groups. The Mahalanobis distance is a descriptive statistic, measuring the distance of a point from a data distribution by calculating the Euclidian distance while taking into account covariance in the data [19]. The matching was implemented using the ‘Matching’ library of the statistical software R [32]. Using a model with replacement means that a single non-conservancy household/individual could be matched with multiple in-conservancy households. In cases where more than one non-conservancy household/individual was tied with a conservancy household as the best match (meaning they were the same Mahalanobis distance away), both of these non-conservancy households/individuals were used and weighted correspondingly.

For each outcome variable, we evaluated which comparison group provided the smallest difference between conservancy and non-conservancy households/individuals in 2000. This was done using a two-sample t-test to compare conservancy households to each of the three non-conservancy comparison groups for each outcome variable. The comparison group that yielded the smallest mean difference when compared to the conservancy group in 2000 was considered to be the best matching model. T-tests were used to ensure that this difference was not statistically different from zero and that the comparison group was therefore not statistically different from the treatment group in 2000 (S2 Table). The best matching model was then applied to the 2006/07 data to produce a comparison group and complete the regression analysis described below. This assumes that because the matching model produced non-statistically different treatment and comparison groups in 2000, when applied in 2006/07 it would produce identical outcomes between treatment and comparison households/individuals in the absence of an impact of conservancies on the outcome variable.

In cases where the inclusion of all matching model variables resulted in a very small set of non-conservancy observations being repeatedly used as matches (resulting in a small and potentially biased set of comparison group observations), some variables were removed from the matching model and instead included as covariates in the post-matching regression model (see below). This resulted in a total of four comparison groups (two different matched comparison groups) being considered to ensure that the best match was found in 2000 (S2 Table). In the case of the wealth index, where the best matching model was unable to match treatment and comparison groups sufficiently in 2000, we modified the matching model using the “MatchBalance” function in the R statistical computing language [33], such that variables that retained significant differences between conservancy and non-conservancy groups post-matching (i.e., a *p*-value less than 10^–16^) were removed. The matching model was thus run instead on this smaller subset of variables. Ultimately, our goal was to minimize the differences between conservancy and non-conservancy comparison groups in 2000, using whatever matching model was able to achieve this.

### Regression Analyses

We used generalized linear models (GLMs) to evaluate the statistical significance of the effects of year and conservancy residence on each outcome variable. A binary logistic regression model was used for all binary outcome variables and a linear regression model for continuous variables:

$$Y_{i}=\beta_{0}+\beta_{1}conservancy_{i}+\beta_{2}year_{i}+\beta_{3}(conservancy_{i}\times year_{i})+\varepsilon_{i}$$ (1)

where Y_i_ is the response variable of the i^th^ household, conservancy_i_ is a binary variable indicating whether the i^th^ household is within a conservancy, year_i_ is a binary variable indicating the year of response by the i^th^ household (if year of survey = 2006/07, year_i_ = 1), β_0_ is the intercept of the regression model, β_1_ and β_2_ are the coefficients on the main effects of conservancy and year, β_3_ is the interaction effect between conservancy and year, and ε_i_ is the error term, which is assumed to be independent and normally distributed. In cases of binary outcome variables, the logit function is:

$$P_{i}=1/(1+e^{-Y})$$ (2)

where P is the probability that the household/individual experiences an increase in the outcome variable, e is a constant, and Y is the log odds of the dependent variable, given by Y_i_ = ln(P_i_/(1-P_i_)). In the case of binary outcome variables, β coefficients in the linear model are interpreted as e^β^ = odds ratio, where the odds ratio expresses the likelihood of change in the dependent variable given a unit of change in the independent variable [34]. In the case of continuous outcome variables, β coefficients in the linear model represent how much the dependent variable is expected to increase when that independent variable increases by one unit, holding all other independent variables constant. The main determinant as to whether conservancy residence has an impact on the temporal trends in each outcome variable was whether the conservancy-year interaction term (β_3_) was significantly different than zero, where a significantly positive coefficient means that the temporal trend in conservancies was significantly greater than the trend in the comparison group.

## Results

### Malaria Prevention

#### Bednet Ownership

**Fig 5:**
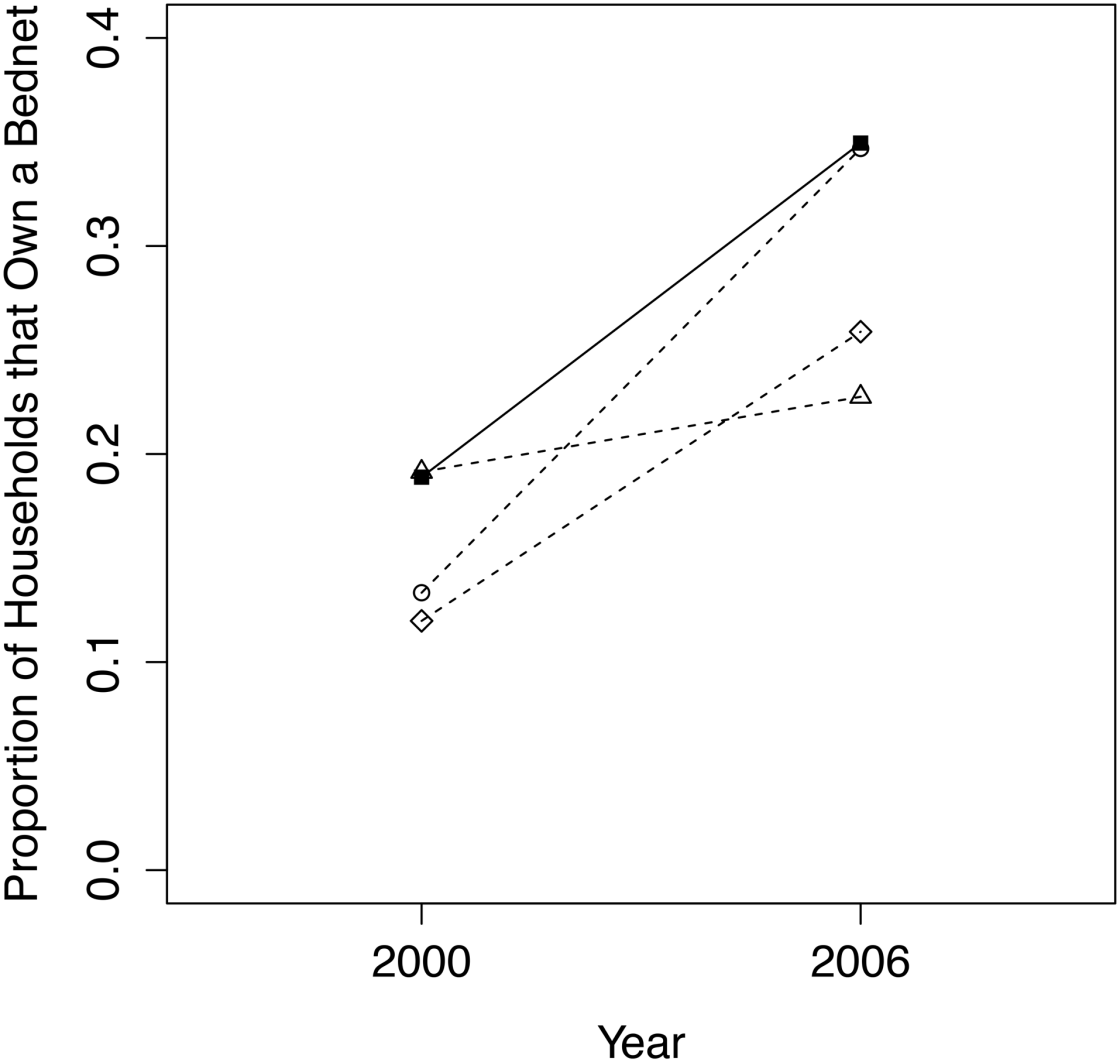
Trends from 2000 to 2006/07 for conservancy households (filled squares, solid line) versus 3 comparison groups (dashed lines, circles = quasi-experimental match; triangles = nearest geographical cluster; diamonds = entire non-conservancy population) for the proportion of households that own a bednet.

In our analysis of household bednet ownership, the comparison group that produced the smallest difference between conservancy and non-conservancy household means in 2000 was the nearest geographical cluster matching model, with a *p*-value of 0.347 (Fig 5). Our final dataset, including all conservancy households and geographically nearest non-residents, contained 750 households in 2000 (400 in conservancy, 350 non-conservancy) and 1170 households in 2006 (581 in conservancy, 589 non-conservancy).

Logistic regression results indicate that conservancy households were significantly more likely to increase the proportion of bednet ownership from 2000 to 2006/07, relative to those in the comparison group (Table 1). Th The odds ratio indicates that in-conservancy households are more than twice as likely to have increased bednet ownership over this time period as non-conservancy households. The second set of regression results reported in Table 1 are produced by a regression model including two additional covariates (education of household head and household wealth) that we expected to have an important effect on the proportion of bednet ownership and which we were interested in observing directly (rather than indirectly via the matching model). The incorporation of these variables improved the pseudo R-squared value of the model and produced similar results, with in-conservancy households being about twice as likely to have increased bednet ownership over time as non-conservancy households. The interaction term remained significant (though not at the same level) indicating that the regression model is robust. When including the covariates, the year effect on bednet ownership becomes stronger and marginally significant, and the conservancy residence coefficient remains insignificant. The education of the household head has a significant and positive effect on bednet ownership, and household wealth is also significant, with a fairly strong negative effect on bednet ownership.

**Table 1:** Logistic regression model results for household bednet ownership.

	Bednet Ownership						
	Coefficient	Std. Error	Z-value	*p*	Odds Ratio	2.5%	97.5%
**No additional covariates**							
Intercept	-1.441	0.136	-10.60	<0.001***	0.237	0.181	0.309
Year	0.218	0.168	1.302	0.193	1.244	0.896	1.728
Conservancy residence	-0.181	0.191	-0.945	0.345	0.835	0.574	1.214
Year:conservancy interaction	0.774	0.232	3.335	<0.001***	2.168	1.376	3.417
	*N* = 1920						
	Pseudo R^2^ = 0.040						
**With additional covariates**							
Intercept	-2.740	0.190	-14.41	<0.001***	0.065	0.044	0.094
Year	0.311	0.177	1.760	0.078^·^	1.364	0.965	1.928
Conservancy residence	-0.135	0.199	-0.680	0.497	0.874	0.592	1.290
Education of household head	0.129	0.014	9.160	<0.001***	1.137	1.106	1.169
Wealth index	-0.998	0.094	-10.60	<0.001***	0.368	0.306	0.443
Year:conservancy interaction	0.687	0.242	2.837	0.005**	1.988	1.236	3.195
	*N* = 1908						
	Pseudo R^2^ = 0.160						

#### Bednet Usage

**Fig 6:**
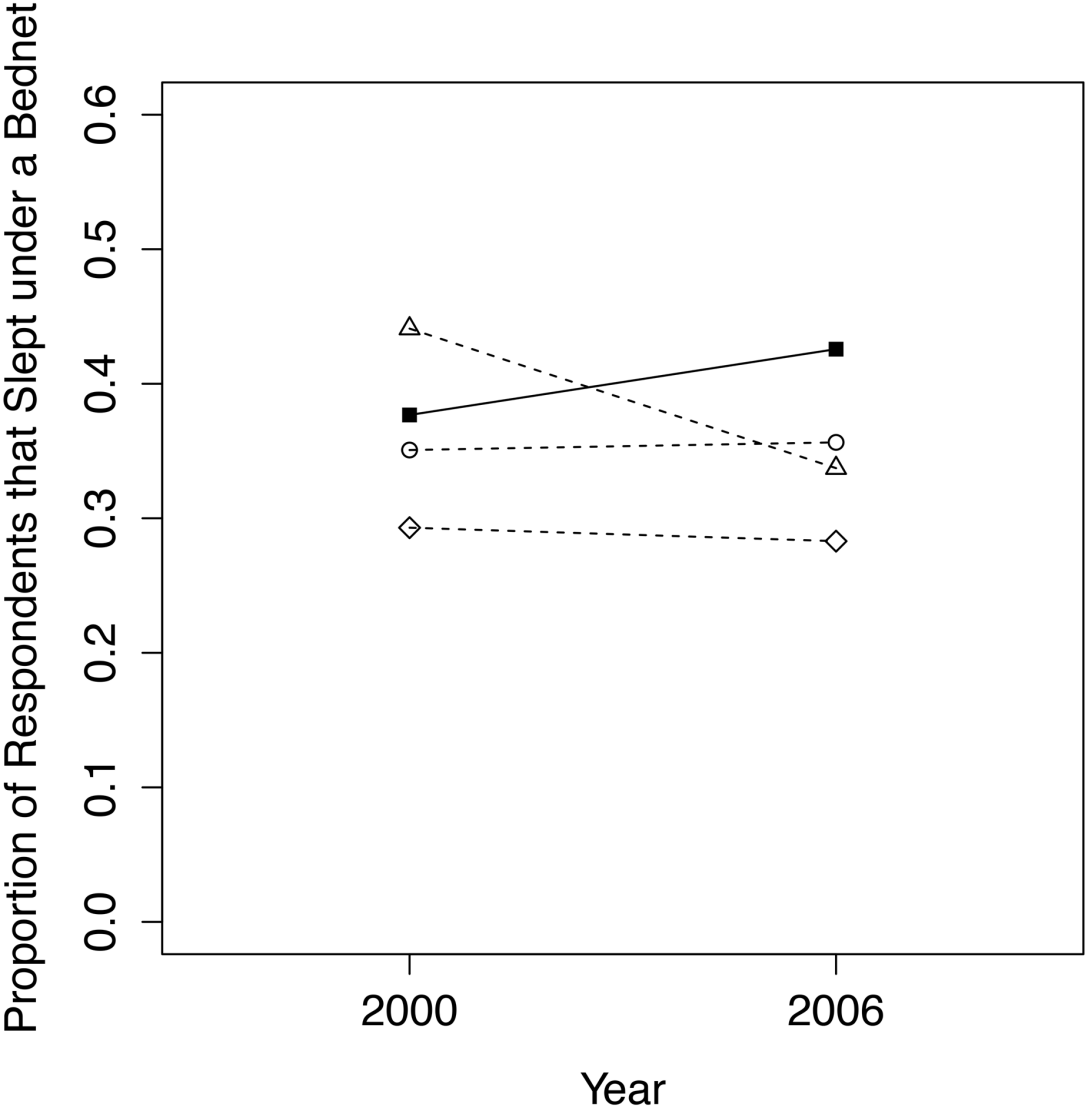
Trends from 2000 to 2006/07 for conservancy residents (filled squares, solid line) versus 3 comparison groups (dashed lines, circles = quasi-experimental match; triangles = nearest geographical cluster; diamonds = entire non-conservancy population) for the proportion of respondents that slept under a bednet during the previous night.

The quasi-experimental matched group was the best comparison group when analyzing the proportion of respondents that slept under a bednet during the previous night, with a *p*-value of 0.754 between treatment and comparison group means in 2000 (Fig 6). Our final dataset, including all in-conservancy female respondents and matched non-conservancy respondents from households that own a bednet, contained 139 women in 2000 (69 conservancy residents, 70 non-conservancy) and 410 women in 2006/07 (202 conservancy residents, 208 non-conservancy).

Although logistic regression results suggest that women in conservancies are approximately 20% more likely than non-conservancy women to have increased bednet usage over time, these results were not in fact statistically significant (Table 2).

**Table 2:** Logistic regression model results for bednet usage by the respondent during the previous night.

	Bednet Usage						
	Coefficient	Std. Error	Z-value	*p*	Odds Ratio	2.5%	97.5%
Intercept	-0.616	0.256	-2.405	0.016*	0.540	0.327	0.892
Year	0.025	0.295	0.084	0.933	1.025	0.575	1.828
Conservancy residence	0.112	0.357	0.316	0.752	1.119	0.556	2.252
Year:conservancy interaction	0.179	0.411	0.435	0.663	1.196	0.534	2.678
	*N* = 547						
	Pseudo R^2^ = 0.006						

### Diarrhea Prevalence and Treatment

#### Diarrhea Prevalence

**Fig 7:**
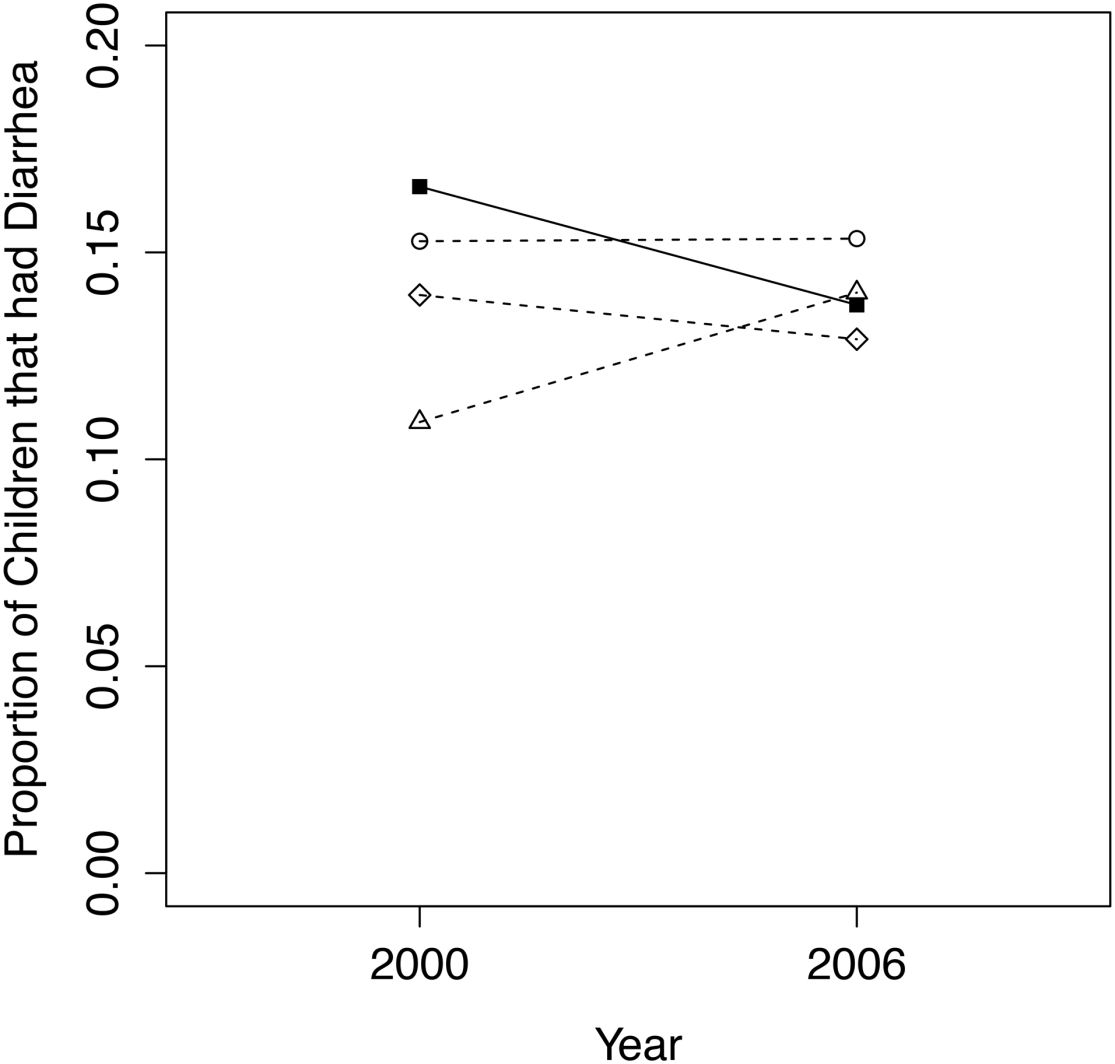
Trends from 2000 to 2006/07 for conservancy children (filled squares, solid line) versus 3 comparison groups (dashed lines, circles = quasi-experimental match; triangles = nearest geographical cluster; diamonds = entire non-conservancy population) for the proportion of children under 5 that had diarrhea within the past 2 weeks.

The comparison group that produced the smallest difference in 2000 between conservancy and non-conservancy children aged 5 and under who experienced diarrhea was the quasi-experimental matched group, with a *p*-value of 0.709 between treatment and comparison group means (Fig 7). Four covariates were removed from the matching model, as their inclusion resulted in a worse match between treatment and comparison groups in 2000. This is caused by the large number of matching variables limiting the number of good matches between conservancy and non-conservancy children in 2000. Variables left out of the matching model were those we were interested in observing the effect of and were included as covariates in the post-matching regression model instead. The final dataset used for the analysis of this variable contained 447 children in 2000 (217 conservancy members, 230 non-conservancy) and 614 children in 2006 (284 conservancy residents, 330 non-conservancy). No significant effects of conservancy residence (or indeed any other variable) on diarrhea experienced in the last two weeks were observed in either the core or expanded covariate regression model (Table 3).

**Table 3:** Logistic regression model results for diarrhea prevalence in children under 5 within the past 2 weeks.

	Diarrhea Prevalence						
	Coefficient	Std. Error	Z-value	*p*	Odds Ratio	2.5%	97.5%
**No additional covariates**							
Intercept	-1.714	0.191	-8.992	<0.001***	0.180	0.124	0.262
Year	0.005	0.249	0.020	0.984	1.005	0.617	1.637
Conservancy residence	0.099	0.264	0.374	0.709	1.104	0.658	1.851
Year:conservancy interaction	-0.228	0.354	-0.644	0.520	0.796	0.398	1.593
	*N* = 1061						
	Pseudo R^2^ = 0.001						
**With additional covariates**							
Intercept	-1.479	0.295	-5.01	<0.001 ***	0.228	0.128	0.406
Year	-0.144	0.264	-0.55	0.585	0.866	0.517	1.452
Conservancy residence	-0.096	0.276	-0.35	0.728	0.908	0.529	1.561
Education of household head	-0.037	0.023	-1.58	0.113	0.964	0.921	1.009
Wealth index	0.086	0.162	0.53	0.596	1.090	0.793	1.498
Distance to health facility	0.000	0.000	0.26	0.797	1.000	1.000	1.001
Improved water source	0.031	0.226	0.14	0.892	1.031	0.663	1.605
Year:conservancy interaction	0.086	0.370	0.23	0.817	1.089	0.528	2.249
	*N* = 995						
	Pseudo R^2^ = 0.006						

#### Diarrhea Treatment

**Fig 8:**
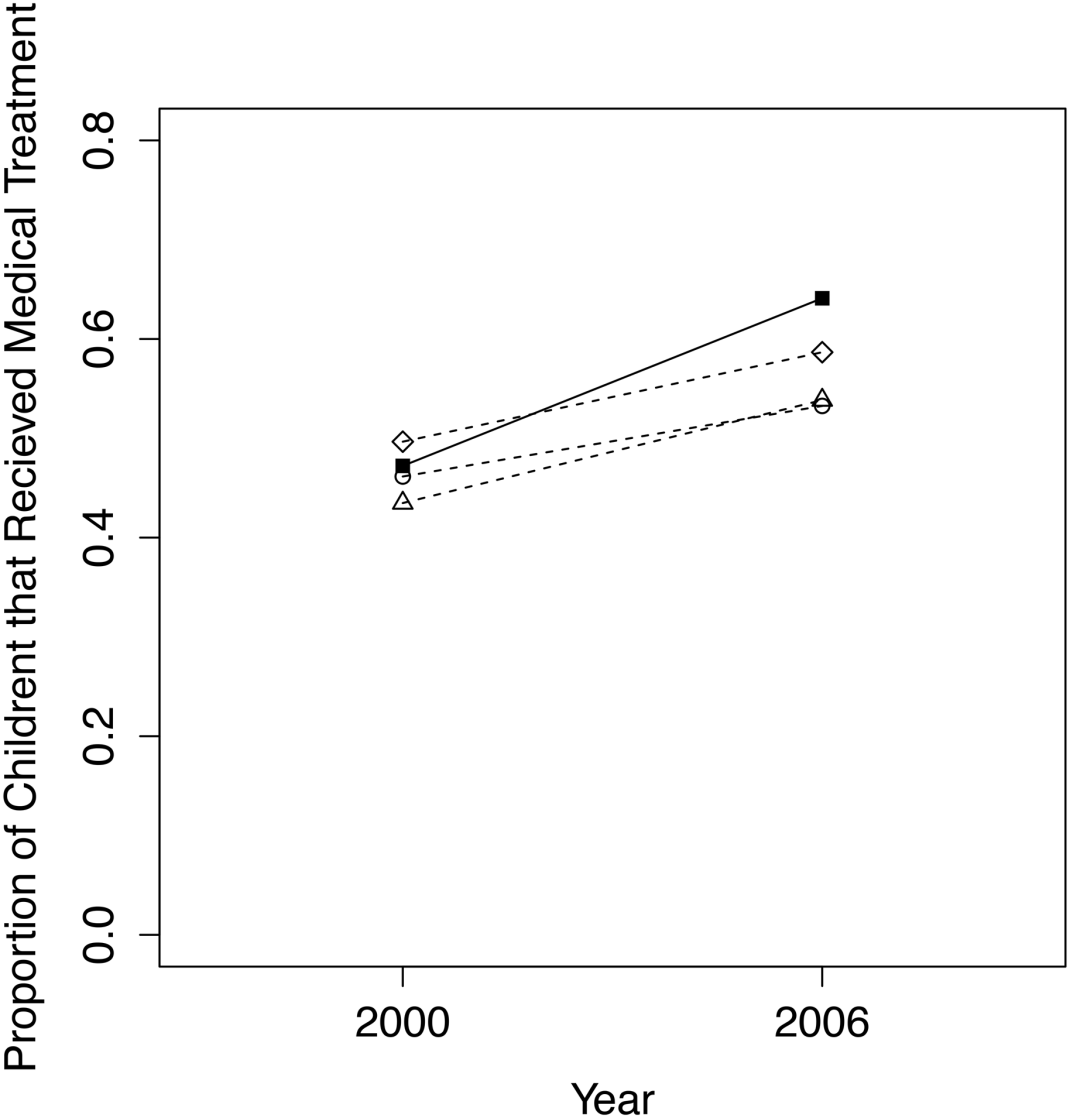
Trends from 2000 to 2006/07 for conservancy children (filled squares, solid line) versus 3 comparison groups (dashed lines, circles = quasi-experimental match; triangles = nearest geographical cluster; diamonds = entire non-conservancy population) for the proportion of children under 5 that received medical treatment for their diarrhea in the past 2 weeks.

The best comparison group for the proportion of children who received medical treatment for diarrhea in the past two weeks was the quasi-experimental matched group, with a *p*-value of 0.931 between treatment and comparison group means in 2000 (Fig 8). Our final dataset, including all conservancy children and matched non-conservancy children, included 69 children in 2000 (36 conservancy residents, 33 non-conservancy) and 89 children in 2006 (39 conservancy residents, 50 non-conservancy). The reason for the small sample size is that only children whose mothers’ responded yes to them having had diarrhea in the past two weeks were eligible to answer this question. Four additional covariates were removed from the matching model, as they weakened the match between conservancy and non-conservancy children in 2000 for the same reasons given for diarrhea prevalence above. As with diarrhea prevalence, regression results show no significant impact on changes over time in diarrhea treatment for children in conservancies compared to non-conservancy children (Table 4). Including additional covariates in the regression model improved the pseudo-R-squared value, but all covariates as well as the interaction term remained insignificant.

**Table 4:** Logistic regression model results for diarrhea treatment in children under 5 within the past 2 weeks.

	Diarrhea Treatment						
	Coefficient	Std. Error	Z-value	*p*	Odds Ratio	2.5%	97.5%
**No additional covariates**							
Intercept	-0.154	0.352	-0.438	0.661	0.857	0.430	1.708
Year	0.285	0.460	0.620	0.535	1.329	0.540	3.272
Conservancy residence	0.043	0.485	0.088	0.929	1.044	0.403	2.701
Year:conservancy interaction	0.406	0.659	0.617	0.537	1.501	0.413	5.460
	*N* = 158						
	Pseudo R^2^ = 0.026						
**With additional covariates**							
Intercept	-0.231	0.571	-0.40	0.686	0.794	0.260	2.430
Year	0.435	0.498	0.87	0.382	1.545	0.582	4.103
Conservancy residence	0.295	0.522	0.57	0.572	1.343	0.483	3.735
Education of household head	0.005	0.046	0.11	0.915	1.005	0.918	1.100
Wealth index	-0.411	0.347	-1.18	0.237	0.663	0.336	1.309
Distance to health facility	-0.002	0.001	-1.63	0.104	0.998	0.996	1.000
Improved water source	-0.242	0.446	-0.54	0.588	0.785	0.327	1.883
Year:conservancy interaction	0.296	0.709	0.42	0.677	1.344	0.335	5.397
	*N* = 144						
	Pseudo R^2^ = 0.090						

### School Attendance

**Fig 9:**
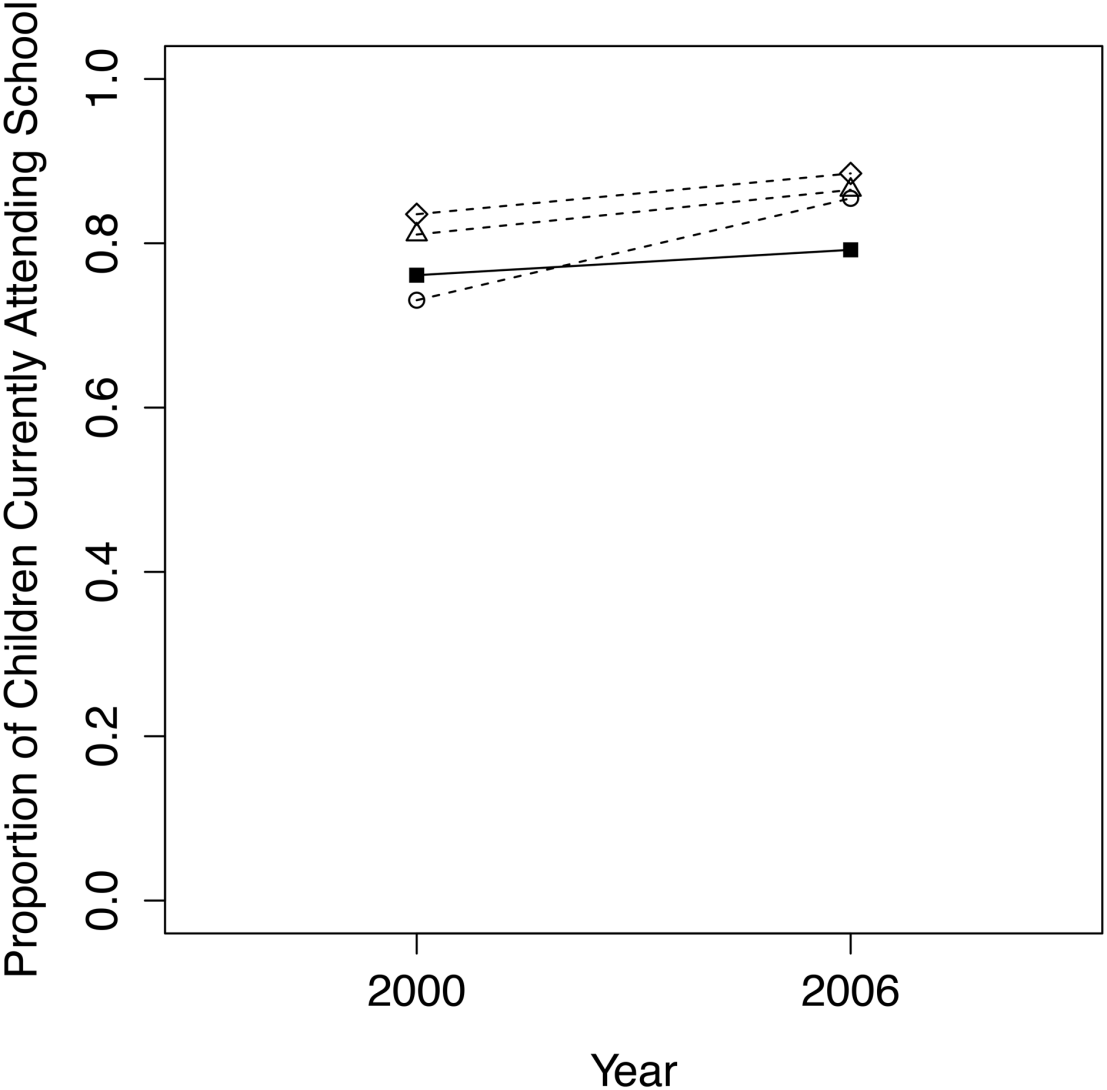
Trends from 2000 to 2006/07 for conservancy children (filled squares, solid line) versus 3 comparison groups (dashed lines, circles = quasi-experimental match; triangles = nearest geographical cluster; diamonds = entire non-conservancy population) for the proportion of school-aged children that attended school during the current year.

In our analysis of school attendance by children (ages 6–16), the best comparison group was the quasi-experimentally matched model, with a *p*-value of 0.307 between conservancy and comparison children in 2000 (Fig 9). Our final dataset contained 877 children in 2000 (427 conservancy residence, 450 non-conservancy) and 1239 children in 2006 (601 conservancy residents, 638 non-conservancy). Logistic regression results indicate that school attendance of conservancy children is stable between 2000 and 2006/07, while school attendance of the matched comparison group increases over time to a significant degree. Conservancy children have a significantly lower rate of growth in school attendance over time, with the odds ratio indicating that the proportion of conservancy children attending school is approximately 45% less likely to increase over time than non-conservancy children (Table 5).

**Table 5:** Logistic regression model results for school attendance of children ages 6–16.

	School Attendance						
	Coefficient	Std. Error	Z-value	*p*	Odds Ratio	2.5%	97.5%
Intercept	0.998	0.109	9.149	<0.001***	2.713	2.191	3.360
Year	0.774	0.159	4.865	<0.001***	2.167	1.587	2.960
Conservancy residence	0.161	0.157	1.021	0.307	1.174	0.863	1.599
Year:conservancy interaction	-0.595	0.220	-2.710	0.007*	0.551	0.359	0.848
	*N* = 2116						
	Pseudo R^2^ = 0.020						

### Household Wealth

**Fig 10:**
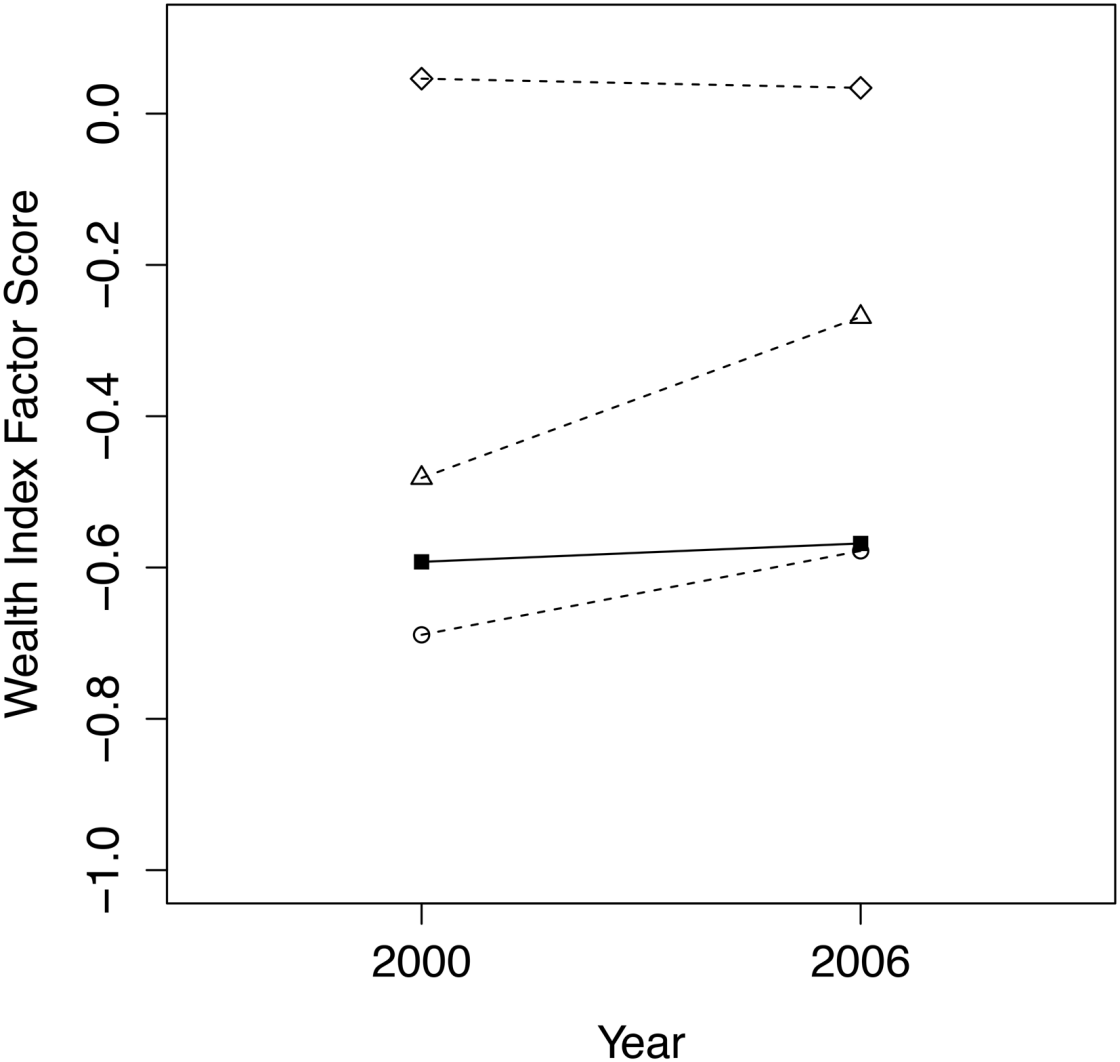
Trends from 2000 to 2006/07 for conservancy households (filled squares, solid line) versus 3 comparison groups (dashed lines, circles = quasi-experimental match; triangles = nearest geographical cluster; diamonds = entire non-conservancy population) for standardized household wealth factor scores.

For household wealth index factor scores, the matching models we tried were unable to reduce mean differences between conservancy and non-conservancy households to a non-statistically significant level in 2000 until certain matching variables were removed from the quasi-experimental matching model. The variables removed were those that showed the least improvement between treatment and comparison groups after the matching model was run (altitude, distance to main roads and precipitation). The results of the matching once these variables were removed is shown in Fig 10, where the quasi-experimental matching model is the best comparison group with a (just) non-statistically significant difference between the two group means (*p*-value = 0.060). In this case, our dataset contained 1032 households in 2000 (289 in conservancy, 743 non-conservancy) and 2484 households in 2006/07 (578 in conservancy, 1906 non-conservancy). Linear regression results (Table 6) indicate that non-conservancy households are significantly more likely to experience an increase in wealth index between 2000 and 2006 as compared to conservancy households.

**Table 6:** Linear regression model results for household wealth index factor scores.

	Wealth Index			
	Coefficient	Std. Error	t-value	*p*
Intercept	-0.689	0.028	-24.874	<0.001***
Year	0.111	0.034	3.276	0.001**
Conservancy residence	0.096	0.039	2.457	0.014*
Year:conservancy interaction	-0.087	0.048	-1.809	0.071
	*N* = 3516			
	Multiple R^2^ = 0.004 Adjusted R^2^ = 0.003			

## Discussion

### Health Outcomes

The presence of the CBNRM program in Namibia has had positive household-level effects on health outcomes related to malaria prevention. Bednet ownership inside conservancies was significantly more likely to increase over time than ownership outside conservancies, and the trend in bednet usage was similar (i.e., usage in conservancies also improved over time relative to the comparison group, although not to a statistically significant degree). Our matching analysis accounted for the effects of wealth and urban/rural residence, as well as geographic location, between conservancy and non-conservancy groups, implying that this finding is not due to targeting of bednet dispersal to rural, low income communities or to a higher risk of malaria in conservancy regions. Increased bednet ownership can therefore be attributed to the presence of conservancies and considered a benefit of the CBNRM program. The community structure provided by conservancies may make it easier for government distribution of bednets and improve the effectiveness of current bednet distribution and education programs [35]. Although there are no specific malaria-prevention education programs associated with conservancies, other health-related education programs such as the HIV/AIDS outreach and education program may encourage conservancy residents to educate themselves and be more active in preventing disease. Training and capacity building programs are provided by tourism enterprises to employees and by NGOs to conservancy staff, and one of the effects of this training may be a better understanding of the importance of disease prevention and treatment, as well as increased confidence of women in sharing their ideas and opinions [17].

Our results indicate that a more educated head of household improves the likelihood of owning a bednet, both in and out of conservancies. This is expected based on previous analysis of bednet ownership in Sub-Saharan Africa [36]. Our results also indicate that wealthier households are less likely to own a bednet, both in and out of conservancies. This is an unexpected result and may be due to three possible reasons: wealthier homes may use different malaria prevention methods such as secure screens, repellent creams and antimalarial medications; bednet distribution and promotion programs may preferentially target lower income households [35]; or this may be due to a structural difference if poorer households live in regions with a higher risk of malaria. Analysis of wealth and malaria distributions in the country suggest that this may be the case, as lower income households and higher risk of malaria both occur in the most northern Namibian provinces [37,38].

The best matching model for bednet usage was the quasi-experimentally matched group, but we note that if the geographically nearest matching model is chosen (which was also not statistically different to the treatment group in 2000; *p*-value = 0.447), the increase in usage over time in conservancies becomes significantly greater than the change outside of conservancies (Fig 8). This demonstrates the importance of matching model selection when determining effects of conservancies over time. All things considered, there is strong evidence for a positive impact of the CBNRM program on malaria prevention behaviour of residents.

Trends in prevalence and treatment of diarrhea in conservancy children were not statistically different from non-conservancy children. Analysis of the diarrhea treatment variable was done using a small sample size, since only a fraction of children were eligible for this question, and may have compromised the power of the statistical analysis to detect differences. Previous analyses in different regions (such as [5]), the expected benefits of ecosystem conservation (such as natural water purification processes) and expected benefits of conservancy structure (such as community taps to provide safe drinking water), all suggest that in theory, Namibia’s CBNRM program could be expected to have a positive effect on diarrhea prevalence in conservancies [21]. Our results were unable to verify this prediction, but the use of a longer time period and larger sample size in future analysis may improve our power to do so.

### Education Outcomes

Our results indicate that school attendance of children living in conservancies is less likely to increase over time compared to non-conservancy children. The school attendance of conservancy children is not decreasing over time, but is rather remaining the same while the school attendance of non-conservancy children increases. Under the assumption that conservancy children would match the school attendance of non-conservancy children in the absence of conservancies, our results suggest that the presence of conservancies is responsible for this lack of improvement in school attendance over time. These results support the findings of [18], which suggest that indirect benefits of the CBNRM program, including improved infrastructure such as schools, have not yet been realized for many communities, although we are unclear as to why our comparison group children appear to be attending school at greater rates than conservancy children.

### Wealth Outcomes

Although previous research has demonstrated community-level financial benefits due to conservancies, it is not clear whether these positive impacts would be expected at the household level [10,15,16]. Our results indicate that the economic benefits derived from conservancies are not affecting the wealth index of individual households, as this measure of wealth changes very little between 2000 and 2006. The wealth of comparison group households was lower in 2000 than that of conservancy households, and while not significant at the 0.05 level, the difference is substantial enough (*p*-value = 0.06) to suspect that the observed relative wealth gains of the comparison group may in fact be due to the lower starting point. Additionally, the small R-squared value of our regression model suggests that many of the factors affecting household wealth are not captured in our best model, leaving room for further studies that are better able to capture this dimension of household welfare.

### Limitations and Assumptions

The use of DHS data means that surveys were designed to be nationally and regionally representative, but were not designed to take into account the location of conservancies. We assumed that the matching of households dealt with this issue of unequal survey distribution inside and outside of conservancies. The DHS are not panel surveys, meaning that the households interviewed in 2006/07 were not the same as in 2000, causing difficulties in comparison between the two years. Our analysis was also limited to the questions asked by the DHS surveys, which inevitably lack some of the confounding factors impacting the outcomes we were interested in.

Furthermore, a limitation of the 2000 DHS survey, in terms of health outcomes, was a lack of relevant variables allowing for analysis of nutrition. Ordinarily our analysis of the health impacts of conservancies would include a nutrition outcome variable, but due to the apparent universal nature of the nutrition-related variables collected in this survey (such as vitamin A supplements), we were unable to draw any conclusions in this regard.

Our analysis assumes that a matching model that produces statistically identical households in 2000 would produce a similar effect in 2006/07 in the absence of conservancies. The validity of this assumption depends on the strength of the matching model used, and improvements in the matching model for certain variables may provide a more robust analysis of socioeconomic trends over time. Additionally, we treated all conservancies as equal in our analysis, whereas in reality, conservancies vary in their age, management effectiveness, inherent biological and socioeconomic conditions, and other factors that could impact the results.

We note that the R-squared and pseudo-R-squared values for most regression models were small, suggesting that the models did not capture all the variability in the data for most outcome variables. Since most outcome variables were binary, the pseudo-R-squared values were not expected to be close to one, but were still low given that a value between 0.2 and 0.4 generally indicates a good fit [34]. We also note that, given the limitations described above, our findings demonstrate only associations, not causal relationships, between conservancy residence and socioeconomic well-being.

## Conclusions

Our results indicate mixed effects of Namibia’s CBNRM program at the household level, given the data available. Relative to appropriately matched comparison groups, we found that Namibia’s conservancy program had a positive effect on malaria prevention, a negative effect on school attendance and household wealth, and no observed effects on diarrhea prevalence. This empirical analysis, while limited in the ways described above, tends to confirm a recent review that suggests highly variable impacts of Namibia’s CBNRM program on households [17]. For community conservation programs such as this one, it is therefore important to analyze many different indicators of livelihoods, at a variety of levels, in order to rigorously evaluate programmatic impacts. As such, we recommend further analysis that extend the impacts we and others have considered. In particular, the forthcoming release of the 2013 DHS surveys, from which an extended time period, more variables, and larger sample sizes can be drawn, will allow for a valuable addition to the current body of work. Use of the 2013 DHS will also capture any impacts of conservancies that have occurred within the past 6 years, which are not captured in analyses to-date. It is important to note that this study did not consider the issue of equity in the distribution of benefits and costs associated with conservancy activities. Future work should address these issues for an improved understanding of the effects of conservancies on Namibians.

## Supporting Information

S1 Table Summary statistics for outcome variables. (DOCX)

S2 Table Summary statistics and t-test results used to determine the best matching model for each outcome variable in 2000. Bolded values indicate the matching model chosen. (DOCX)
