# Supplementary material for: Effects of Community-Based Natural Resource Management on Household Welfare in Namibia
Source: PLoS One. 2015 May 12;10(5):e0125531. doi: 10.1371/journal.pone.0125531 (PMC4429124; doi:10.1371/journal.pone.0125531)
Supplement: S1 Table — (DOCX) [file pone.0125531.s001.docx]

**Table S1.** Summary statistics for outcome variables.

|  | | | **In conservancy** | | | | | **Outside conservancy** | | | | |
| --- | --- | --- | --- | --- | --- | --- | --- | --- | --- | --- | --- | --- |
| **Year** | **Variable** | **Best Matching Model** | **Obs** | **Mean** | **Std. Dev.** | **Min** | **Max** | **Obs^1^** | **Mean** | **Std. Dev.** | **Min** | **Max** |
| **2000** | **Bednet ownership** | Geographically nearest | 400 | 0.165 | 0.372 | 0 | 1 | 350 | 0.191 | 0.394 | 0 | 1 |
|  | **Bednet usage** | Matched (with covariates) | 69 | 0.377 | 0.488 | 0 | 1 | 67 | 0.351 | 0.481 | 0 | 1 |
|  | **Diarrhea prevalence** | Matched (no covariates) | 217 | 0.166 | 0.373 | 0 | 1 | 212.8 | 0.153 | 0.361 | 0 | 1 |
|  | **Diarrhea treatment** | Matched (no covariates) | 36 | 0.472 | 0.506 | 0 | 1 | 32.5 | 0.462 | 0.506 | 0 | 1 |
|  | **School attendance** | Matched (with covariates) | 427 | 0.761 | 0.427 | 0 | 1 | 427 | 0.731 | 0.444 | 0 | 1 |
|  | **Wealth index** | Matched (no covariates; precipitation, altitude, & distance to main roads removed) | 289 | -0.593 | 0.694 | -1.147 | 1.706 | 289 | -0.689 | 0.523 | -1.110 | 1.485 |
| **2006/07** | **Bednet ownership** | Geographically nearest | 581 | 0.348 | 0.477 | 0 | 1 | 589 | 0.228 | 0.420 | 0 | 1 |
|  | **Bednet usage** | Matched (with covariates) | 202 | 0.426 | 0.496 | 0 | 1 | 202 | 0.356 | 0.480 | 0 | 1 |
|  | **Diarrhea prevalence** | Matched (no covariates) | 284 | 0.137 | 0.345 | 0 | 1 | 300 | 0.153 | 0.361 | 0 | 1 |
|  | **Diarrhea treatment** | Matched (no covariates) | 39 | 0.641 | 0.486 | 0 | 1 | 46 | 0.533 | 0.504 | 0 | 1 |
|  | **School attendance** | Matched (with covariates) | 601 | 0.792 | 0.406 | 0 | 1 | 602 | 0.855 | 0.353 | 0 | 1 |
|  | **Wealth index** | Matched (no covariates; precipitation, altitude & distance to main roads removed) | 578 | -0.568 | 0.771 | -1.452 | 1.720 | 578 | -0.578 | 0.615 | -1.452 | 1.639 |

^1^Values reported for matched comparison groups are weighted according to the output from ‘Matching’ in R.
