# Supplementary material for: Effects of Community-Based Natural Resource Management on Household Welfare in Namibia
Source: PLoS One. 2015 May 12;10(5):e0125531. doi: 10.1371/journal.pone.0125531 (PMC4429124; doi:10.1371/journal.pone.0125531)
Supplement: S2 Table — (DOCX) [file pone.0125531.s002.docx]

**Table S2.** Summary statistics and t-test results used to determine the best matching model for each outcome variable. Bolded values indicate the matching model chosen.

|  | | **In Conservancy** | | **Out of Conservancy** | |  | |
| --- | --- | --- | --- | --- | --- | --- | --- |
|  |  | **Mean** | **Variance** | **Mean** | **Variance** | **Mean difference** | ***p*-value** |
| **Bednet ownership** | Matched (no covariates) | 0.189 | 0.154 | 0.109 | 0.098 | -0.080 | 0.009 |
|  | Matched (with covariates) | 0.189 | 0.154 | 0.133 | 0.116 | -0.056 | 0.079 |
|  | Nearest | 0.165 | 0.138 | 0.191 | 0.155 | **0.026** | **0.347** |
|  | All | 0.165 | 0.138 | 0.120 | 0.105 | -0.045 | 0.018 |
| **Bednet usage** | Matched (no covariates) | 0.377 | 0.238 | 0.326 | 0.223 | -0.051 | 0.536 |
|  | Matched (with covariates) | 0.377 | 0.238 | 0.351 | 0.231 | **-0.026** | **0.754** |
|  | Nearest | 0.377 | 0.238 | 0.441 | 0.250 | 0.064 | 0.447 |
|  | All | 0.377 | 0.238 | 0.293 | 0.207 | -0.084 | 0.170 |
| **Diarrhea prevalence** | Matched (no covariates) | 0.166 | 0.139 | 0.153 | 0.130 | **-0.013** | **0.709** |
|  | Matched (with covariates) | 0.150 | 0.128 | 0.133 | 0.116 | -0.017 | 0.639 |
|  | Nearest | 0.166 | 0.139 | 0.109 | 0.098 | -0.057 | 0.087 |
|  | All | 0.166 | 0.139 | 0.140 | 0.120 | -0.026 | 0.316 |
| **Diarrhea treatment** | Matched (no covariates) | 0.472 | 0.256 | 0.462 | 0.256 | **-0.011** | **0.931** |
|  | Matched (with covariates) | 0.500 | 0.259 | 0.800 | 0.167 | 0.300 | 0.019 |
|  | Nearest | 0.472 | 0.256 | 0.435 | 0.257 | -0.037 | 0.783 |
|  | All | 0.472 | 0.256 | 0.497 | 0.251 | 0.024 | 0.782 |
| **School attendance** | Matched (no covariates) | 0.761 | 0.182 | 0.794 | 0.164 | 0.033 | 0.243 |
|  | Matched (with covariates) | 0.761 | 0.182 | 0.731 | 0.197 | **-0.030** | **0.307** |
|  | Nearest | 0.757 | 0.184 | 0.811 | 0.154 | 0.054 | 0.025 |
|  | All | 0.757 | 0.184 | 0.835 | 0.138 | 0.079 | 0.000 |
| **Wealth index** | Matched (no covariates) | -0.593 | 0.481 | -0.705 | 0.283 | -0.113 | 0.029 |
|  | Matched (with covariates) | -0.593 | 0.481 | -0.703 | 0.272 | -0.111 | 0.031 |
|  | Matched (no covariates; precipitation, altitude & distance to main roads removed) | -0.593 | 0.481 | -0.689 | 0.274 | **0.096** | **0.060** |
|  | Nearest | -0.642 | 0.401 | -0.482 | 0.676 | 0.160 | 0.002 |
|  | All | -0.642 | 0.401 | 0.046 | 1.011 | 0.688 | 0.000 |
